# Diagnostic performance of ultrasonography-based risk models in differentiating between benign and malignant ovarian tumors in a Chinese cohort

**DOI:** 10.3389/fonc.2026.1914278

**Published:** 2026-10-14

**Authors:** Weimei Li, Ziwei Sun, Qin Huang, Junhong Zhou, Yanming Li, Ying Gao, Meng Li

**Affiliations:** 1 Department of Ultrasound in Medicine, Shanghai Sixth People’s Hospital Affiliated to Shanghai Jiao Tong University School of Medicine; Shanghai Institute of Ultrasound in Medicine, Shanghai, China; 2 Shanghai Key Laboratory of Neuro-Ultrasound for Diagnosis and Treatment, Shanghai, China; 3 Department of Pathology, Shanghai Sixth People’s Hospital Affiliated to Shanghai Jiao Tong University School of Medicine, Shanghai, China; 4 Department of Cardiology, QingPu Branch of Zhongshan Hospital Affiliated to Fudan University, Shanghai, China

**Keywords:** ADNEX model, IOTA simple rules, O-RADS model, ovarian tumor, ultrasound

## Abstract

**​​Background:**

Ultrasound-based risk models provide a valuable tool to support non-expert clinicians in evaluating adnexal masses, potentially reducing unnecessary surgeries for benign conditions. However, their implementation in clinical practice across China remains suboptimal, and direct comparisons of their diagnostic accuracy within this specific population are still needed.

**​Objective:**

To evaluate and compare the diagnostic performance of three ultrasonography-based risk models—the International Ovarian Tumor Analysis (IOTA) Simple Rules (with expert adjudication for inconclusive cases), the IOTA Assessment of Different Neoplasias in the Adnexa (ADNEX) model, and the Ovarian-Adnexal Reporting and Data System (O-RADS)—for distinguishing benign from malignant adnexal lesions in a Chinese cohort.

**Methods:**

This retrospective diagnostic study was conducted at a single Chinese academic medical center and included consecutive patients aged 18 to 89 years with adnexal masses that were managed surgically between January 2023 and December 2024. Each adnexal lesion was systematically evaluated using the IOTA Simple Rules, ADNEX model, and O-RADS classification. The diagnostic performance of these models was quantified by the area under the receiver operating characteristic curve (AUC), sensitivity, specificity, positive and negative predictive values, and likelihood ratios, using surgical pathology as the reference standard. A secondary analysis evaluated the models’ performance stratified by menopausal status. An additional analysis was to evaluate the supplementary value of tumor markers for the O-RADS model.

**​Results:**

A total of 459 female patients with a 15.5% malignant tumor prevalence were included. Mean age was 43.0 (15.2) years overall, with 40.7 (14.4) in benign and 55.3 (13.3) in malignant cases. Overall, 121 (26.4%) patients were postmenopausal. The AUCs for discriminative performance of the ADNEX and O-RADS models were 0.95 (95%CI, 0.93-0.97) and 0.96 (95%CI, 0.94-0.97), respectively. After converting the ADNEX continuous individualized risk into the discrete ordinal categories of O-RADS, the ADNEX performance was reduced to an AUC of 0.92 (95%CI, 0.90-0.95). The AUC of the Simple Rules was 0.95 (95%CI, 0.93-0.97) and yielded a sensitivity of 95.8% (95%CI, 88.1%-99.1%), specificity of 94.3% (95%CI, 91.5%-96.4%). For a binary comparison of all models (benign vs malignant), a uniform 10% risk threshold (O-RADS score of 4-5) was used. The sensitivity and NPV of all models were above 91% and 98%, respectively. The O-RADS model exhibited the highest sensitivity (97.2%; 95%CI, 90.2%-99.7%), whereas the Simple Rules model demonstrated superior specificity 94.3% (95%CI, 91.5%-96.4%) and the highest PPV (75.6%; 95%CI, 65.4%-84.0%). The sensitivities were higher for menopausal groups, while the specificities were higher for premenopausal patients for the ADNEX and O-RADS model. The addition of tumor markers did not improve the diagnostic sensitivity or specificity of the O-RADS model.

**​Conclusion:**

In this diagnostic study of a Chinese cohort, the Simple Rules, ADNEX, and O-RADS models demonstrated effective performance in distinguishing benign from malignant adnexal lesions, consistent with previous reports from European and American populations. The use of such risk-stratification models can enhance the accuracy and reproducibility of adnexal mass assessment, particularly for non-specialist clinicians, and may support clinical decision-making and potentially reduce​ avoidable surgical interventions.

## Introduction

Adnexal masses are common, accounting for 35% of premenopausal women and 17% of postmenopausal women (1). Although the incidence of ovarian cancer is low, it is one of the most lethal gynecological malignancies and ranks as the fifth leading cause of cancer-related mortality in women globally, particularly in those aged 40 and above (2). The cornerstone of improving survival rates lies in accurate preoperative diagnosis, which allows for optimal surgery and direct management by gynecologic oncologists, thereby significantly improving patient prognosis (3, 4). If imaging can consistently indicate a very low risk of malignancy, the tumor can be safely managed with expectant monitoring or handled by general gynecologists (5, 6).

Over the past decades, the International Ovarian Tumor Analysis (IOTA) group has developed and validated several evidence-based ultrasound risk prediction models to standardize the characterization of ovarian tumors. These include the IOTA Simple Rules (SR), which categorize tumors as benign, malignant, or inconclusive based on five benign and five malignant features (7, 8) and the more sophisticated IOTA Assessment of Different Neoplasias in the Adnexa (ADNEX) model, which uses clinical and ultrasound variables to calculate the absolute risk of malignancy and can also differentiate between subtypes of malignant tumors (9, 10). Based on the standardized terminology and data sets from the IOTA group, the American College of Radiology (ACR) introduced the Ovarian-Adnexal Reporting and Data System (O-RADS). Its lexicon was published in 2018 (11), and the full risk stratification system, which also provides specific management recommendations for ovarian tumors, followed in 2020 (12). Despite this, the two primary IOTA models have not been widely adopted, and the newly established O-RADS system also has limited application due to its relatively recent introduction (11–13). The application of these models has been relatively delayed in China. Although the 2024 Chinese national expert consensus now recommends IOTA SR, ADNEX, and O-RADS as first-line tools (recommendation class 2A) and calls for standardized training (14), their integration into routine clinical practice remains limited. While numerous studies conducted in European and American populations have demonstrated the diagnostic advantages of the models for ovarian tumors (15–17), research is ongoing to validate their application value within the Chinese population.

Although serum cancer antigen 125 (CA-125) and human epididymis protein 4 (HE4) are routinely used tumor markers in ovarian cancer evaluation, their incremental value beyond ultrasound-based risk models remains inconsistent—the ADNEX model retains essentially the same discrimination with or without CA-125 (pooled AUC ≈ 0.93 in 47 external validations) (18), and adding HE4 yields only a modest gain (19), while the original ADNEX development cohort already incorporated CA-125 as a core predictor (10). In Chinese cohorts, single-center studies show O-RADS combined with CA-125 and HE4 improves AUC versus O-RADS alone (20). However, to our knowledge, no study has systematically compared all three major risk stratification systems (IOTA SR, ADNEX, and O-RADS) and evaluated the incremental value of tumor markers when added to O-RADS within the same Chinese cohort. Therefore, the present study aimed to: (1) validate and compare the diagnostic performance of the IOTA SR, ADNEX model, and O-RADS system in a Chinese surgical cohort; (2) evaluate the agreement between these models in risk stratification; and (3) assess whether adding CA-125 and HE4 to the O-RADS model provides clinically meaningful improvement in diagnostic accuracy.

## Methods

This was a retrospective single-center diagnostic accuracy study at a Chinese medical center. The study was approved by the Ethics Committee of Shanghai Sixth People’s Hospital [No. 2025-KY-105(K)]. The initial ethics application was submitted on March 27, 2025, and final approval was granted on March 31, 2025, with the official stamped approval letter obtained accordingly. Data collection and analysis commenced in April 2025. The requirement for informed consent was waived due to the retrospective nature of the study. This study involved a review of sonograms from consecutive patients diagnosed with adnexal masses between January 2023 and December 2024. These imaging findings were correlated with prospectively collected clinicopathologic data. All patients had a surgical intervention within 1–2 weeks. Exclusion criteria included pregnancy, being younger than 18 years or older than 89 years, and having pathologically confirmed ovarian cancer (recurrent or previously treated with chemotherapy). Individuals with ultrasonographical normal ovaries, even when findings included follicles or corpus luteum cysts smaller than 3 cm, were also excluded. Postmenopausal status was defined as amenorrhea for ≥ 12 months. For women with prior hysterectomy, age ≥ 50 years was used as a surrogate. CA-125 levels were measured based on clinical indication, and when available, these values were incorporated into the ADNEX model to reflect a real-world clinical setting. CA-125 levels were available for 457 of 459 patients (99.6%). For the 2 cases with missing CA-125 values, the ADNEX model was applied using only the available ultrasound variables, as the model is designed to handle missing predictor values. In addition, some patients also underwent HE4, Carcinoembryonic Antigen (CEA), and Carbohydrate Antigen 19-9 (CA 19-9) testing. Note that CEA and CA 19–9 were collected as part of routine clinical workup but were not included in the primary analysis for the third objective, as they are not established markers for ovarian cancer risk stratification. Their descriptive statistics are presented in **eTable 2** for completeness.

Of the 459 patients, CA-125 was available in 457 (99.6%) and HE4 in 358 (78.0%). Therefore, analyses involving HE4 or the combined CA-125 + HE4 model were based on the subset of 358 patients with complete tumor marker data. Analyses involving CA-125 alone were based on 457 patients.

### Sonographic assessments

Sonographic examinations were performed at the Shanghai Sixth People’s Hospital by experienced sonographers. All scans followed standardized protocols in accordance with the IOTA guidelines. High-end ultrasonography machines (the Samsung Elite WS80A) with both transvaginal (5–9 MHz) and transabdominal (1–5 MHz) probes were used. Images and video clips were stored digitally for subsequent review and risk classification. Scans were systematically reviewed using the well-defined IOTA terms and definitions by two experienced experts (J.H.Z and Z.W.S), who were blinded to the pathological diagnoses and the assessment of each other. In cases of disagreement, a more experienced expert (W.M.L), with more than 15 years of experience in gynecologic ultrasound, made the final assessment. All lesions were assessed using the IOTA SR (7), the IOTA ADNEX model (10), and the ACR O-RADS model, version 1 [lexicon 2018 (11), full system 2020 (12)] (**Figure 1**). For the ADNEX model, the ‘center type’ variable was set to ‘non-oncology center’ as our institution is a general hospital without a dedicated oncology referral center designation. ADNEX predicted risk was categorized into four ordinal groups corresponding to O-RADS categories: <1% (O-RADS 2 equivalent), 1% to 10% (O-RADS 3 equivalent), 10% to 50% (O-RADS 4 equivalent), and >50% (O-RADS 5 equivalent). If a patient had multiple adnexal masses, the mass with the most suspicious morphologic structures was evaluated for statistical analysis. For lesions classified as inconclusive by the SR, W.M.L reviewed the scans and made the final assessment. Of the 459 lesions, 23 (5.0%) initially yielded an ‘inconclusive’ result under the SR. These cases were subsequently reviewed by W.M.L and reclassified as benign or malignant according to overall morphological assessment.

**Figure 1:**
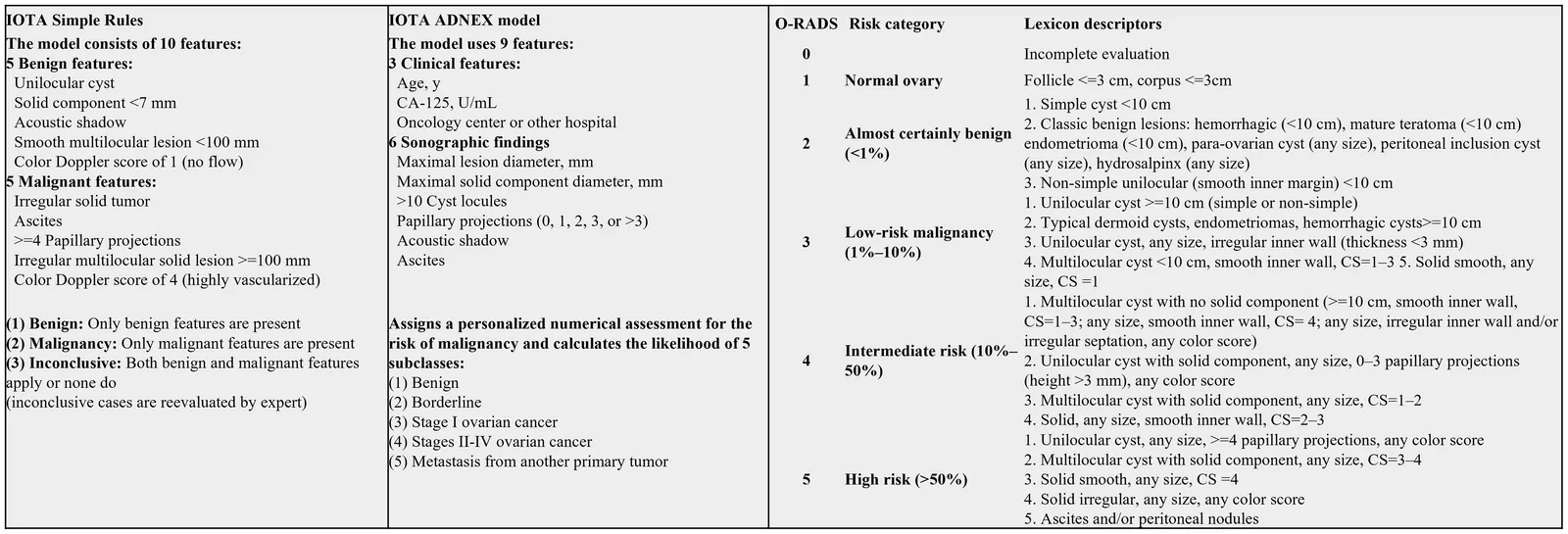
Ultrasonography-based risk models to evaluate patients with adnexal masses. ADNEX, assessment of different neoplasias in the adnexa; CA-125, cancer antigen 125; IOTA, international ovarian tumor analysis; and O-RADS, ovarian-adnexal reporting and data system.

### Statistical analysis

Continuous variables are presented as means and standard deviations (SDs) in addition to medians and interquartile ranges (IQRs). Comparison of demographic and disease characteristics between groups was performed using the *t* test or Mann-Whitney *U* test. Categorical variables are presented as absolute numbers and percentages, and the χ2 test or Fisher exact test was used to compare the groups. Inter-rater agreement between the two independent reviewers for O-RADS categorization was assessed using linear weighted Cohen’s kappa (κ). The κ value was 0.95 (95% CI, 0.92–0.97), indicating almost perfect agreement. To evaluate the diagnostic performance of each model in distinguishing between benign and malignant adnexal lesions, we calculated sensitivity, specificity, positive predictive value (PPV), and negative predictive value (NPV) along with their corresponding 95% confidence intervals (CIs). The area under the receiver operating characteristic (ROC) curve (AUC) was also estimated with 95% CIs, and both positive and negative likelihood ratios were reported. The McNemar test was used to compare sensitivities and specificities between methods. Comparisons of AUCs between methods were performed using the DeLong test. For CA-125 and HE4, ROC curves were generated to evaluate their standalone discriminative performance for distinguishing benign from malignant adnexal masses. The optimal cutoffs were determined using the Youden index (maximizing sensitivity + specificity − 1). Stratified analyses were performed for premenopausal and postmenopausal subgroups. In a sensitivity analysis, the conventional clinical thresholds (CA-125 > 35 U/mL; HE4 > 105.1 pmol/L) were also applied. To assess the robustness of the tumor marker findings to the choice of cutoff values, a sensitivity analysis was performed using conventional clinical thresholds: CA-125 > 35 U/mL and HE4 > 105.1 pmol/L. These thresholds were applied to dichotomize CA-125 and HE4 into binary variables (elevated vs. not elevated). Logistic regression models combining O-RADS categories with CA-125 and/or HE4 status were constructed, and the AUC of each combined model was compared with that of O-RADS alone using the DeLong test. Subgroup analyses were performed stratified by menopausal status. Statistical analysis was performed using SPSS version 20.0 (SPSS Inc, Chicago, Illinois). All tests were 2-sided, and P < 0.05 was considered statistically significant. For statistical analysis, borderline ovarian tumors were included in the malignant group.

## Results

The two independent reviewers demonstrated excellent agreement in O-RADS classification, with a linear weighted kappa of 0.95 (95% CI, 0.92–0.97). Disagreements occurred in 13 of 459 cases (2.8%) and were resolved by consensus with a third senior reviewer. (**eTable 1** in **Supplement 1**).

### Clinical, demographic, sonographic, and pathologic characteristics

A total of 459 Chinese patients with ovarian tumors were included in the final analysis (**efigure 1** in **Supplement 1**). The mean (SD) age of the patients was 43.0 (15.2) years overall. Mean (SD) ages of patients with benign and malignant masses were 40.7 (14.4) and 55.3 (13.3) years, respectively. Overall, 121 (26.4%) patients were postmenopausal. The patients with malignant masses showed higher CA-125 [median (IQR), 22.5(12.1-87.2) U/mL vs. 18.0(11.5-35.7) U/mL], HE4 [median (IQR), 67.4(49.6-98.1) pmol/L vs. 45.5(39.9-55.3) pmol/L], and CEA [median (IQR), 1.6(0.8-3.0) ng/mL vs. 1.1(0.7-1.6) ng/mL] (**eTable 2** in **Supplement 1**).

The median malignant lesion diameter was 89 (58-136) mm, while benign lesions were significantly smaller [52 (38-71) mm]. The presence of solid components [64 (90.1%) vs 44 (11.3%)] and the median maximal solid diameter [median (IQR), 47 (29-65) mm vs. 22 (10-35) mm)] were significantly greater in malignant lesions compared with benign lesions. Malignant lesions were also more likely to have more than 10 locules [13 (18.3%) vs. 13 (3.4%)], more than 3 papillary projections [8 (11.3%) vs. 1 (0.3%)], more presence of ascites [32 (45.1%) vs. 2 (0.5%)], more presence of acoustic shadow [17 (23.9%) vs. 3 (0.8%)], and higher vascular scores [35 (49.3%) vs. 1 (0.3%)] on Doppler assessment, compared with benign lesions (**eTable 3** in **Supplement 1**).

According to histopathology, 388 (84.5%) tumors were benign, 9 (2%) were borderline, and 62 (13.5%) were malignant. Overall, the most common benign pathology was endometrioma, and the most common malignant pathology was serous cystadenocarcinoma. In the premenopausal group [338 (73.6%)], the most common benign lesions were endometrioma [116 (34.3%)] and mature teratoma [76(22.5%)], and the most common malignant lesions was serous cystadenocarcinoma [10(3.0%)]. In the postmenopausal group [121 (26.4%)], the most common benign lesions were serous cystadenoma [24 (19.8%)] and mucinous cystadenoma [21 (17.4%)], and the most common malignant lesions were serous cystadenocarcinoma [18(14.9%)] and mucinous cystadenocarcinoma (6.6%) (**eTable 4** in **Supplement 1**).

### Risk models and diagnostic performance

We tested the diagnostic performance of the following ultrasonography-based risk models: SR, ADNEX, and O-RADS (**Figure 1**).

CA-125 levels were available for 457 of 459 patients (99.6%). Analysis of the ADNEX model’s performance revealed a statistically significant difference in the median calculated risk of malignancy between benign lesions [median (IQR), 3.3 (2.4-4.7) %] and malignant lesions [median (IQR), 53.6 (25.1-85.1) %] (P < 0.001) (**eTable 5** in **Supplement 1**). The model’s ability to discriminate between these groups was excellent, with an area under the ROC curve (AUC) of 0.95 (95% CI, 0.93-0.97) (**Figure 2**). Using the 10% probability threshold as a cutoff for malignancy, the model yielded a sensitivity of 91.6% (95% CI, 82.5%-96.8%), a specificity of 88.1% (95% CI, 84.5%-91.2%), a positive predictive value (PPV) of 58.6%, and a negative predictive value (NPV) of 98.3% (**Table 1**). At a lower threshold of 5%, the sensitivity increased to 97.2% (95% CI, 90.2%-99.7%) with a specificity of 77.3% (95% CI, 72.8%-81.4%) (**eTable 6** in **Supplement 1**).

**Figure 2:**
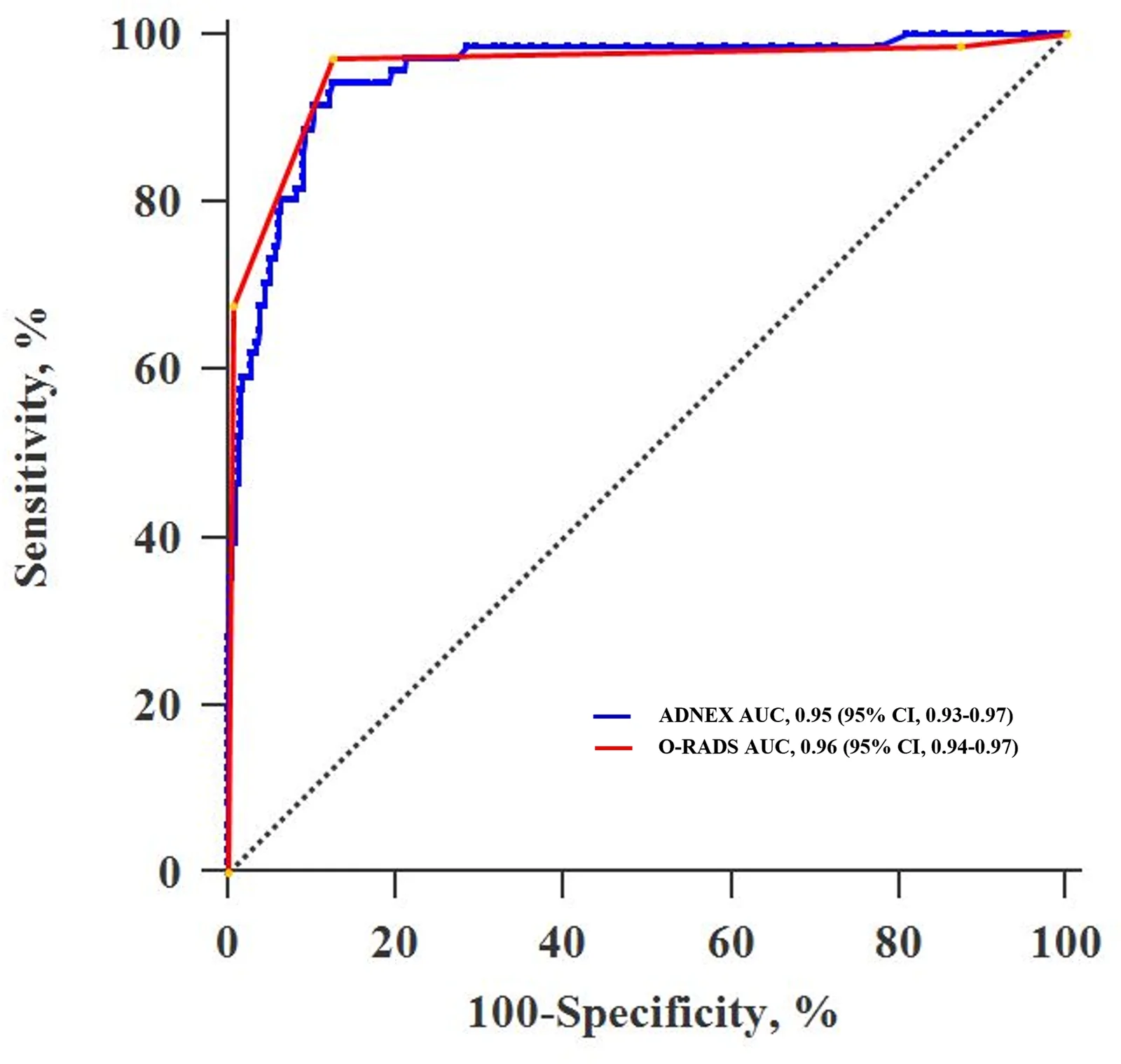
Receiver operating characteristic (ROC) curves for the diagnostic performance of the Assessment of Different Neoplasias in the Adnexa (ADNEX) and Ovarian-Adnexal Reporting and Data System (O-RADS) models The ADNEX model assigns a personalized numerical assessment for the risk of malignant tumor (continuous risk, 0%-100%). The O-RADS model classifies each lesion into 1 of 6 risk categories with a score of 0 to 5: 0 for incomplete evaluation and 1 for normal ovary including physiologic cyst; hence, the ROC curve analysis shown includes O-RADS risk scores of 2 to 5 (ordinal categories that correlate with 0%-100% risk of malignant tumor). AUC indicates area under the ROC curve.

**Table 1:** Diagnostic performance of different ultrasonography-based risk models among 459 patients.

Risk model	Sensitivity, % (95% CI)	Specificity, % (95% CI)	PPV, % (95% CI)	NPV, % (95% CI)	Positive LR, % (95% CI)	Negative LR, % (95% CI)
Simple Rules	95.8(88.1-99.1)	94.3(91.5-96.4)	75.6(65.4-84.0)	99.2(97.6-99.8)	16.9(16.0-17.8)	0.05(0.01-0.1)
ADNEX model with cutoff at 10%	91.6(82.5-96.8)	88.1(84.5-91.2)	58.6(48.8-67.8)	98.3(96.3-99.4)	7.7(7.1-8.4)	0.1(0.04-0.2)
O-RADS model, category 2–3 vs 4-5	97.2(90.2-99.7)	87.4(83.6-90.5)	58.5(49.0-67.5)	99.4(97.9-99.9)	7.7(7.3-8.1)	0.03(0.008-0.1)

ADNEX, Assessment of Different Neoplasias in the Adnexa; LR, likelihood ratio; NPV, negative predictive value; O-RADS, Ovarian-Adnexal Reporting and Data System; PPV, positive predictive value.

After applying the SR, 349 cases (76.0%) were classified as benign, 87 cases (19.0%) as malignant, and 23 cases (5.0%) as inconclusive. After expert adjudication, the 23 inconclusive cases were reclassified as 20 benign and 3 malignant, yielding the final distribution used for diagnostic performance analysis. The ROC curve analysis for the overall AUC of the SR approach to differentiate between benign and malignant masses was 0.95 (95%CI, 0.93-0.97) and yielded a sensitivity of 95.8% (95%CI, 88.1%-99.1%), specificity of 94.3% (95%CI, 91.5%-96.4%) (**Table 1**).

Using the O-RADS model, the observed malignant tumor frequencies in O-RADS categories 2, 3, 4, and 5 were 2.0% (1 of 50), 0.3% (1 of 291), 31.3% (21 of 67), and 94.1% (48 of 51), respectively (**eTable 5, 7** in **Supplement 1**). The ROC curve analysis for the overall performance of O-RADS showed an AUC of 0.96 (95%CI, 0.94-0.97) (**Figure 2**). At O-RADS categories 4-5, the sensitivity, specificity, PPV, and NPV were 97.2% (95%CI, 90.2%-99.7%), 87.4% (95%CI, 83.6%-90.5%), 58.5% (95%CI, 49.0%-67.5%), and 99.4% (95%CI, 97.9%-99.9%), respectively (**Table 1**).

### Comparison of risk models

The overall diagnostic performance of the ADNEX and O-RADS models was analyzed using the ROC curve analysis (**Figure 2**). Since the ADNEX model provides a continuous risk score while the O-RADS and SR are based on discrete ordinal categories, a direct comparison of their AUCs is not methodologically sound. To enable a fair comparison, the continuous ADNEX risk scores were converted into ordinal categories corresponding to O-RADS scores 2 through 5 (**Figure 1**). After this conversion, the overall performance of the ADNEX model decreased from an AUC of 0.95 (95% CI, 0.93–0.97) to 0.92 (95% CI, 0.90–0.95). The difference between the converted ADNEX and O-RADS (AUC, 0.96; 95% CI, 0.94–0.97) was statistically significant (P < 0.001, DeLong test) (**Figure 3**). The observed malignant tumor frequencies using the ADNEX model to stratify patients into O-RADS categories 2, 3, 4, and 5 were 0% (0 of 4), 1.7% (6 of 343), 39.7% (27 of 68), and 86.3% (38 of 44), respectively (**eTable 7** in **Supplement 1**). Both models demonstrated increasing malignancy rates with higher risk categories, confirming their discriminative ability.

**Figure 3:**
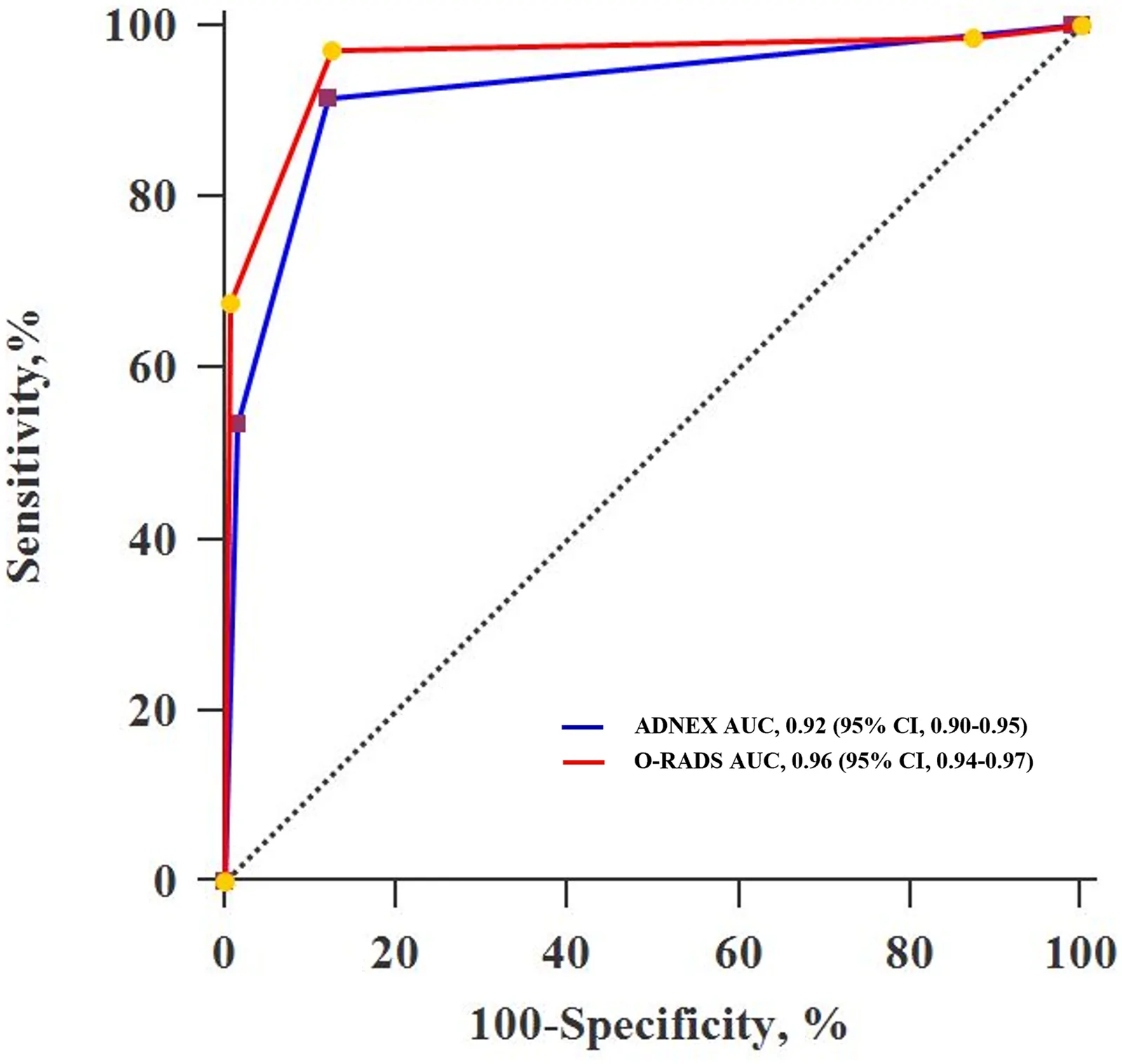
Receiver operating characteristic (ROC) curves for the diagnostic performance of ovarian-adnexal reporting and Data System (O-RADS) and Assessment of Different Neoplasias in the Adnexa (ADNEX) models using discrete ordinal categories The O-RADS model classifies each lesion into 1 of 6 risk categories with a score of 0 to 5: 0 for incomplete evaluation and 1 for normal ovary including physiologic cyst; hence, the ROC curve analysis shown includes O-RADS risk scores of 2 to 5 (ordinal categories that correlate with 0%-100% risk of malignant tumor). Converting the ADNEX continuous personalized risk of malignant tumor (0%-100%) into discrete ordinal categories as defined by the O-RADS model (scores 2-5) resulted in a reduced overall diagnostic performance of the ADNEX model; the area under the curve (AUC) decreased from 0.95 to 0.92. The difference between the converted ADNEX and O-RADS (AUC, 0.96; 95% CI, 0.94–0.97) was statistically significant (P < 0.001, DeLong test).

For a binary comparison of all models (benign vs malignant), a uniform 10% risk threshold (O-RADS score of 4-5) was used. The sensitivity and NPV of all models were above 91% and 98%, respectively (**Table 1**). The O-RADS model exhibited the highest sensitivity (97.2%; 95%CI, 90.2%-99.7%), whereas the SR model demonstrated superior specificity (94.3%, 95%CI, 91.5%-96.4%) and the highest PPV (75.6%; 95%CI, 65.4%-84.0%); In contrast, the O-RADS model had the lowest specificity (87.4%; 95%CI, 83.6%-90.5%) and PPV (58.5%; 95%CI, 49.0%-67.5%) (**Table 1**). The observed malignancy rates by O-RADS category were generally consistent with the expected ranges: O-RADS 2 (2.0%, expected ~0%), O-RADS 3 (0.3%, expected <1%), O-RADS 4 (31.3%, expected 1–50%), and O-RADS 5 (94.1%, expected >50%). Notably, the malignancy rate in O-RADS 5 (94.1%) fell within the expected range (>50%), confirming the high specificity of this category for malignancy.

### Tumor markers

As detailed in Methods, analyses for HE4 and CA-125 were performed on the available subsets of 358 and 457 patients, respectively. ROC analysis was performed to evaluate the discriminative performance of CA-125 and HE4 as standalone biomarkers. The optimal cutoffs derived from the Youden index were: CA-125 overall > 126.4 U/mL (AUC 0.58, 95% CI 0.53-0.63), premenopausal > 138.3 U/mL (AUC 0.54, 95% CI 0.48-0.59), postmenopausal > 13.6 U/mL (AUC 0.76, 95% CI 0.67-0.83); HE4 overall > 55.7 pmol/L (AUC 0.75, 95% CI 0.70-0.79), premenopausal > 55.7 pmol/L (AUC 0.66, 95% CI 0.60-0.72), postmenopausal > 65.2 pmol/L (AUC 0.73, 95% CI 0.63-0.81) (**eTable 8** in **Supplement 1**). Of note, within the ADNEX model, CA-125 was entered as a continuous variable (log-transformed) per the original model specification, rather than using a dichotomized threshold.

Using ROC-derived optimal cutoffs (CA-125 > 126.4 U/mL; HE4 > 55.7 pmol/L), adding CA-125 alone to O-RADS yielded an AUC of 0.957 (Δ = +0.005, P = 0.303), adding HE4 alone yielded an AUC of 0.956 (Δ = +0.003, P = 0.557), and adding both markers yielded an AUC of 0.960 (Δ = +0.008, P = 0.076). None of these improvements reached statistical significance, indicating that neither CA-125 nor HE4, alone or in combination, provided meaningful incremental diagnostic value beyond O-RADS.

Sensitivity analysis using conventional clinical thresholds (CA-125 > 35 U/mL; HE4 > 105.1 pmol/L) confirmed the robustness of the primary findings. The addition of CA-125 alone, HE4 alone, or both markers to O-RADS yielded no statistically significant improvement in AUC (0.961, 0.955, and 0.953, respectively; all P > 0.38), with absolute AUC changes ranging from −0.003 to +0.004.

### Subgroups and sensitivity analyses

Subgroup analysis stratified by menopausal status revealed consistent patterns. In premenopausal women (n=338, 28 malignant), the AUC of O-RADS was 0.93 (95% CI, 0.89–0.98) and ADNEX was 0.93 (95% CI, 0.87–0.98). In postmenopausal women (n=121, 43 malignant), the AUC of O-RADS was 0.96 (95% CI, 0.94–0.99) and ADNEX was 0.97 (95% CI, 0.93–0.99) (**Table 2**).

**Table 2:** Diagnostic performance of different ultrasonography-based risk models by menopausal status.

Outcome by subgroup	Simple Rules combined with expert evaluation for inconclusive cases	ADNEX model with cutoff at 10%	O-RADS model, category 2–3 vs 4-5
Premenopausal women			
Sensitivity, % (95% CI)	89.3(71.8-97.7)	85.7(67.3-96.0)	92.9(76.5-99.1)
Specificity, % (95% CI)	99.7(98.2-100)	90.6(86.8-93.6)	88.7(84.6-92.0)
PPV, % (95% CI)	96.2(79.9-99.9)	45.3(31.4-59.7)	42.6(30.0-55.9)
NPV, % (95% CI)	99.0(97.2-99.8)	98.6(96.4-99.6)	99.3(97.4-99.9)
Postmenopausal women			
Sensitivity, % (95% CI)	93.0(80.9-98.5)	95.3(94.2-99.4)	100(91.8-100)
Specificity, % (95% CI)	100(95.4-100)	79.5(68.8-87.8)	82.1(71.7-89.8)
PPV, % (95% CI)	100(91.0-100)	71.9(58.5-83.0)	75.4(62.2-85.9)
NPV, % (95% CI)	96.3(89.6-99.2)	96.9(89.1-99.6)	100(94.4-100)

ADNEX, Assessment of Different Neoplasias in the Adnexa; NPV, negative predictive value; O-RADS, Ovarian-Adnexal Reporting and Data System; PPV, positive predictive value.

Regarding tumor markers, CA-125 alone had poor performance in premenopausal women (AUC 0.54; 0.48 -0.59), but improved markedly in postmenopausal women (AUC 0.76; 0.67 -0.83). In contrast, HE4 showed more consistent performance across both groups, with AUCs of 0.66 (0.60 -0.72) and 0.73 (0.63 -0.81), respectively. In premenopausal women, adding CA-125 and/or HE4 to O-RADS yielded no meaningful AUC improvement (all Δ ≤ 0.005). Similarly, in postmenopausal women, AUC improvements were negligible (all Δ ≤ 0.003). These findings confirm that the lack of incremental value of tumor markers was consistent across both menopausal subgroups. (**eTable 8** in **Supplement 1**).

## ​Discussion​

In this Chinese surgical cohort, all three ultrasound-based risk stratification systems, IOTA SR, ADNEX, and O-RADS, demonstrated high diagnostic accuracy for differentiating benign from malignant adnexal masses, with AUC values ≥ 0.95 and negative predictive values exceeding 98% across all models. It should be noted, however, that NPV is dependent on disease prevalence; despite the relatively higher malignancy rate in our cohort (15.5% vs. ~10% in unselected populations (5)), the NPV remained above 98% across all models, reinforcing the high NPV observed for these systems in this cohort. These findings indicate that any of the three systems showed high NPV in this Chinese surgical cohort, supporting their use for preoperative risk assessment. Although O-RADS showed a statistically significant advantage over the converted ADNEX model (AUC 0.96 vs. 0.92; P < 0.001), the absolute difference of 0.04 is unlikely to alter clinical decision-making. Furthermore, adding CA-125 and/or HE4 to O-RADS yielded no statistically significant improvement in diagnostic accuracy, regardless of whether ROC-derived cutoffs or conventional clinical thresholds were used (all ΔAUC ≤ 0.008, all P > 0.05), suggesting that routine measurement of these biomarkers may not provide additional diagnostic benefit when structured ultrasound reporting systems are available.

These findings are broadly consistent with prior international and Chinese validation studies. The overall AUC for the ADNEX model in this study (0.95) is well within the range of 0.91 to 0.97 reported in previous studies (9, 10, 21–25). For the O-RADS model, our AUC of 0.96 falls within the wider documented range of 0.89 to 0.98 (12, 16, 23, 26–29). For the IOTA SR, the reported sensitivity and specificity in prior validation studies range from 80% to 97% and 69% to 99%, respectively (7, 8, 30, 31). In our cohort, the SR achieved a sensitivity of 95.8% and specificity of 94.3%, consistent with the upper end of these reported ranges. Previous studies have suggested that for cases deemed inconclusive by the SR, evaluation by experienced experts is more appropriate than automatically classifying them as malignant (15). Therefore, in this study, such cases were directly adjudicated by an expert to determine whether they were benign or malignant, and the results were consistent with those reported in prior research (15, 30–32).

To our knowledge, this is one of the largest single-center evaluations comparing all three risk stratification systems in a Chinese cohort. Our study exclusively enrolled surgically managed patients, whereas several previous IOTA and O-RADS validation cohorts included both surgically and conservatively managed cases. Despite this difference in cohort selection, our findings corroborate their key conclusion: all models demonstrated high sensitivity (>91%) and notable variation in specificity and PPV, with the SR achieving the highest values, followed by the ADNEX and O-RADS models. This observation also aligns with several studies in Chinese cohorts (23, 25, 33), which reported comparable diagnostic performance between the ADNEX and O-RADS models, with both demonstrating high AUC values exceeding 0.90. Crucially, all models maintained a NPV exceeding 98%, indicating a high NPV following a negative test result in this cohort. When comparing ultrasound-based risk models, a trade-off between sensitivity and specificity is inevitable. This balance is not a technical flaw but stems from the inherent characteristics of different models and diverse clinical needs. The choice of which balance strategy to adopt depends on a range of complex factors (34–36).

To further compare the diagnostic performance among the models, we adopted the methodology proposed by Yoeli-Bik et al (15), which discretized the ADNEX continuous personalized risk of malignancy into ordinal categories comparable to the O-RADS scores. Regarding the comparison between O-RADS and the converted ADNEX model, although the DeLong test showed a statistically significant difference (AUC 0.96 vs. 0.92; P < 0.001), the absolute difference was only 0.04. In the context of ovarian cancer risk stratification, where both models already demonstrate excellent discriminative performance (AUC ≥ 0.90), such a small difference is unlikely to translate into clinically meaningful changes in patient management. Both models achieved a NPV exceeding 98%, indicating that either showed a high NPV in this cohort. Therefore, the choice between O-RADS and ADNEX may depend more on local expertise, workflow preferences, and familiarity with each system rather than on marginal differences in AUC.

In this study, optimal cutoffs for CA-125 and HE4 were derived from ROC curves using the Youden index to maximize discriminative performance within our cohort. This approach provides an estimate of the best-case standalone performance of these biomarkers. CA-125 alone had poor overall discriminative ability (AUC 0.58), largely driven by its weak performance in premenopausal women (AUC 0.54; 0.48 -0.59) versus moderate performance in postmenopausal women (AUC 0.76; 0.67 -0.83). HE4 showed moderate overall performance (AUC 0.75), with similar AUCs between premenopausal (0.66; 0.60 -0.72) and postmenopausal groups (0.73; 0.63 -0.81). The notably lower CA-125 cutoff in postmenopausal women (13.6 U/mL) compared with premenopausal women (138.3 U/mL) reflects the known physiological elevation of CA-125 in premenopausal women due to benign conditions such as endometriosis and menstruation, which reduces its specificity in this population. Conversely, the higher HE4 cutoff in postmenopausal women (65.2 pmol/L vs. 55.7 pmol/L) is consistent with age-related increases in HE4 levels.

When CA-125 and HE4 (dichotomized at the cohort-derived cutoffs) were added to O-RADS using logistic regression, the combined AUC increased from 0.952 to 0.960 (Δ = +0.008, P = 0.076). Neither the ROC-derived cutoff analysis nor the sensitivity analysis using conventional clinical thresholds (all P > 0.38) showed statistically significant improvement, confirming the limited incremental value of these biomarkers beyond O-RADS. This finding aligns with previous studies reporting limited incremental value of serum biomarkers beyond ultrasound-based risk models (18, 19). Several factors may explain this lack of meaningful improvement. First, the ultrasound features captured by O-RADS and ADNEX—such as solid components, papillary projections, and ascites—are biologically correlated with the same pathological processes that drive CA-125 and HE4 elevation, resulting in substantial feature redundancy (9, 18). Second, the near-perfect NPV of O-RADS alone (>98%) leaves minimal room for improvement by adding biomarkers (23). Third, within the ADNEX model, CA-125 is already incorporated as a continuous predictor; therefore, evaluating its incremental value outside the model is conceptually redundant (9).

It should be noted that the cutoffs used in the primary analysis were cohort-derived and may not generalize to other populations. The use of ROC-derived optimal cutoffs may yield optimistic performance estimates compared with fixed clinical thresholds. Nonetheless, the sensitivity analysis using conventional thresholds confirmed the robustness of our conclusions. External validation is warranted to confirm these findings in broader populations.

### Limitations

This study has several limitations. First, the exclusive inclusion of surgically treated patients introduces verification and spectrum bias. Conservatively managed benign masses were excluded, resulting in an artificially elevated malignancy prevalence (15.5% vs. approximately 10% in unselected populations (5)), which may overestimate sensitivity and specificity. Second, CA-125 was unavailable for 2 of 459 patients (0.4%); although the missing rate was low, the heterogeneity in data availability for the ADNEX model was not fully addressed. Third, the reported performance of the IOTA SR reflects a hybrid workflow combining algorithmic rules with expert adjudication of inconclusive cases (5.0%), rather than the rules in isolation. Fourth, the thresholds used to discretize ADNEX continuous risk into ordinal categories (<1%, 1–10%, 10–50%, >50%) were cohort-specific and have not been externally validated; the resulting AUC may vary with different cutoff choices. Fifth, the tumor marker analysis may be underpowered to detect small incremental effects, given the limited number of malignant cases (n=71). Sixth, this study used O-RADS version 1 (2018–2020); the updated O-RADS v2022 may yield different results. Seventh, borderline ovarian tumors (n=9, 1.9%) were grouped with malignant tumors for analysis; although this is consistent with prior studies, borderline tumors have distinct biological behavior and prognosis. Finally, this was a single-center retrospective study without external validation; prospective multicenter studies are warranted to confirm our findings.

## Conclusion​

In this Chinese surgical cohort, the IOTA SR, ADNEX model, and O-RADS system all demonstrated high diagnostic accuracy for differentiating benign from malignant adnexal masses, with AUC values ≥ 0.95 and NPV exceeding 98% across all models. Although O-RADS showed a statistically significant advantage over the converted ADNEX model (AUC 0.96 vs. 0.92; P < 0.001), the absolute difference was small (0.04) and unlikely to be clinically meaningful. Both models performed consistently well across premenopausal and postmenopausal subgroups, supporting their broad applicability. Adding CA-125 and HE4 to O-RADS did not yield a statistically significant improvement, suggesting that routine measurement of these biomarkers may not provide additional diagnostic benefit when structured ultrasound reporting systems are available. These findings support the clinical utility of these systems for preoperative risk assessment, with the choice depending primarily on local expertise and workflow preferences. However, prospective multicenter studies with external validation and inclusion of conservatively managed patients are needed to confirm these findings in unselected populations.

## Data Availability

The raw data supporting the conclusions of this article will be made available by the authors, without undue reservation.
