# Supplementary material for: Diagnostic performance of ultrasonography-based risk models in differentiating between benign and malignant ovarian tumors in a Chinese cohort
Source: Front Oncol. 2026 Oct 14;16:1914278. doi: 10.3389/fonc.2026.1914278 (PMC13713523; doi:10.3389/fonc.2026.1914278)
Supplement: Supplementary file 1 [file Table1.docx]

**eFigure 1. Flowchart Illustrating the Patient Selection and Inclusion in the Final Cohort**

| **eTable 1. Cross-Tabulation of O-RADS Assignments Between Two Independent Reviewers** | | | | | |
| --- | --- | --- | --- | --- | --- |
|  | **Reviewer 2: O-RADS 2** | **Reviewer 2: O-RADS 3** | **Reviewer 2: O-RADS 4** | **Reviewer 2: O-RADS 5** | **Total** |
| **Reviewer 1: O-RADS 2** | 48 | 2 | 0 | 0 | 50 |
| **Reviewer 1: O-RADS 3** | 1 | 287 | 3 | 0 | 291 |
| **Reviewer 1: O-RADS 4** | 0 | 4 | 61 | 2 | 67 |
| **Reviewer 1: O-RADS 5** | 0 | 0 | 1 | 50 | 51 |
| **Total** | 49 | 293 | 65 | 52 | 459 |

Linear weighted kappa = 0.95 (95% CI, 0.92–0.97)

| **eTable 2. Demographics and Clinical Characteristics of Patients with Benign and Malignant Adnexal Masses^a^** | | | | |
| --- | --- | --- | --- | --- |
| **Demographic and clinical characteristics** | **All patients (n=459)** | **Benign  (n=388)** | **Malignant  (n=71)** | **p-value** |
| **Age (years)** |  |  |  |  |
| Age [mean (±SD)] | 43.0(±15.2) | 40.7(±14.4) | 55.3(±13.3) |  |
| Age [median (IQR)] | 41(32-52) | 38(31-49) | 56(46-64) | <0.001^c^ |
| **Menopausal status** |  |  |  |  |
| Premenopausal | 338(73.6%) | 310(79.9%) | 28(39.4%) | <0.001^d^ |
| Postmenopausal | 121(26.4%) | 78(20.1%) | 43(60.6%) |  |
| **CA-125 (U/mL)** |  |  |  |  |
| CA-125 available | 457(99.6%) | 387(99.7%) | 70(98.5%) |  |
| CA-125 level [mean (±SD)] | 59.2(±170.5) | 38.0(±59.2) | 175.9(±395.3) |  |
| CA-125 level [median (IQR)] | 18.5(11.5-41.9) | 18.0(11.5-35.7) | 22.5(12.1-87.2) | 0.033^c^ |
| CA-125 elevated (>35 U/mL) | 126(27.6%) | 99(25.6%) | 27(38.6%) | 0.025^d^ |
| **HE4 (pmol/L)** |  |  |  |  |
| HE4 available | 358(78.0%) | 296(76.3%) | 62(87.3%) |  |
| HE4 level [mean (±SD)] | 73.8(±147.3) | 55.4(±87.5) | 162.0(±283.7) |  |
| HE4 level [median (IQR)] | 47.2(40.1-58.8) | 45.5(39.9-55.3) | 67.4(49.6-98.1) | <0.001^c^ |
| HE4 elevated (>105.1 pmol/L) | 20(5.6%) | 7(2.4%) | 13(21.0%) | <0.001^d^ |
| **CEA (ng/mL)** |  |  |  |  |
| CEA available | 451(98.3%) | 383(98.7%) | 68(95.8%) |  |
| CEA level [mean (±SD)] | 1.7(±3.5) | 1.3(±0.8) | 3.9(±8.4) |  |
| CEA level [median (IQR)] | 1.2(0.7-1.7) | 1.1(0.7-1.6) | 1.6(0.8-3.0) | <0.001^c^ |
| CEA elevated (>5 ng/mL) | 11(2.4%) | 2(0.5%) | 9(13.2%) | <0.001^e^ |
| **CA-199 (U/mL)** |  |  |  |  |
| CEA available | 445(96.9%) | 377(97.2%) | 68(95.8%) |  |
| CEA level [mean (±SD)] | 26.7(±71.9) | 25.1(±69.9) | 35.7(±82.1) |  |
| CEA level [median (IQR)] | 13.0(6.5-22.4) | 13.1(6.5-22.7) | 12.4(6.0-21.6) | 0.973^c^ |
| CEA elevated (>37 U/mL) | 54(12.1%) | 45(11.9%) | 9(13.2%) | 0.763^d^ |

^a^For continuous variables, data are means (± standard deviation, SD) and medians (interquartile range, IQR). For categorical variables, data are the number of patients (column %). P-values are obtained by ^b^t-test or ^c^Mann-Whitney U-test for continuous variables; ^d^chi-square or ^e^Fisher’s exact test for categorical variables, as appropriate.

CA-125, Carbohydrate Antigen 125; HE4, Human Epididymis Protein 4; CEA, Carcinoembryonic Antigen; CA-199, Carbohydrate Antigen 19-9

| **eTable 3. Sonographic Characteristics of Patients with Benign and Malignant Adnexal Masses^a^** | | | | |
| --- | --- | --- | --- | --- |
| **Sonographic characteristics** | **All patients  (n=459 )** | **Benign (n=388)** | **Malignant (n=71)** | **p-value** |
| **Lesion type by IOTA terminology** | | | | |
| Unilocular | 300(65.4%) | 298(76.8%) | 2(2.8%) | <0.001^d^ |
| Unilocular solid | 27(5.9%) | 18(4.6%) | 9(12.7%) |  |
| Multilocular | 51(11.1%) | 46(11.9%) | 5(7.0%) |  |
| Multilocular solid | 31(6.8%) | 11(2.8%) | 20(28.2%) |  |
| Solid | 50(10.9%) | 15(3.9%) | 35(49.3%) |  |
| **Sonographic variables*** | | | | |
| Maximal lesion diameter (mm) [mean (±SD)] | 67.0(±42.2) | 60.1(±34.2) | 104.6(±58.8) |  |
| Maximal lesion diameter (mm) [median (IQR)] | 55(40-80) | 52(38-71) | 89(58-136) | <0.001^e^ |
| Presence of solid component | 108(23.5%) | 44(11.3%) | 64(90.1%) | <0.001^c^ |
| Maximal solid diameter (mm) [mean (±SD)] | 38.9(±26.2) | 25.6(±21.4) | 49.6(±24.9) |  |
| Maximal solid diameter (mm) [median (IQR)] | 35(20-55) | 22(10-35) | 47(29-65) | <0.001^e^ |
| More than ten locules | 26(5.7%) | 13(3.4%) | 13(18.3%) | <0.001^c^ |
| Four or more papillary projections | 9(2.0%) | 1(0.3%) | 8(11.3%) | <0.001^d^ |
| Presence of ascites | 34(7.4%) | 2(0.5%) | 32(45.1%) | <0.001^d^ |
| Presence of acoustic shadow | 20(4.4%) | 3(0.8%) | 17(23.9%) | <0.001^d^ |
| **Color score**^b^ | | | | |
| Color score 1 (no flow) | 370(80.6%) | 359(92.5%) | 11(15.5%) | <0.001^d^ |
| Color score 2 (minimal flow) | 53(11.5%) | 28(7.2%) | 25(35.2%) |  |
| Color score 3 (moderate flow) | 26(5.7) | 1(0.3%) | 25(35.2%) |  |
| Color score 4 (strong flow) | 10(2.2%) | 0(0) | 10(14.1%) |  |

^a^The lesions were systematically reviewed using a standardized protocol based on well-defined IOTA terms and definitions. Most (85%) sonographic examinations were performed at the University of Chicago; the minority of scans were conducted at affiliated facilities and were reviewed through the PACS system. For continuous variables, data are both means (± standard deviation, SD) and medians (interquartile range, IQR). For categorical variables, data are the number of patients (column %).

^b^Color score is based on the IOTA terms and is defined by subjective assessment of blood flow in the lesions (scores 1-4).

P-values are obtained by ^c^chi-square or ^d^Fisher’s exact for categorical variables, as appropriate; and ^e^Mann-Whitney U-test for continuous variables.

| **eTable 4. Histopathologic Findings of 459 adnexal masses** | | | |
| --- | --- | --- | --- |
| **Tumor pathology** | **All cases (n=459)** | **Premenopausal women (n=338)** | **Postmenopausal women (n=121)** |
| **Benign tumors** |  |  |  |
| Endometrioma | 119(25.9%) | 116(34.3%) | 3(2.5%) |
| Mature teratoma | 81(17.6%) | 76(22.5%) | 5(4.1%) |
| Cystadenofibroma | 5(1.1%) | 2(0.6%) | 3(2.5%) |
| Serous cystadenoma | 58(12.6%) | 34(10.1%) | 24(19.8%) |
| Mucinous cystadenoma | 45(9.8%） | 24(7.1%) | 21(17.4%) |
| Epidermoid cyst | 4(0.9%) | 3(0.9%) | 1(0.8%) |
| Hemorrhagic cyst, hemorrhagic corpus luteum | 25(5.4%) | 23(6.8%) | 2(1.7%) |
| Para-ovarian simple cyst | 34(7.4%) | 21(6.2%) | 13(10.7%) |
| Fibroma, thecoma, fibrothecoma | 9(2.0%) | 3(0.9%) | 6(5.0%) |
| Struma ovarii | 1(0.2%) | 1(0.3%) | 0(0) |
| Tubo-ovarian abscess, pyosalpinx, hematosalpinx | 2(0.4%) | 2(0.6%) | 0(0) |
| Other benign lesions^a^ | 5(1.1%) | 5(1.5%) | 0(0) |
| **Borderline tumors** |  |  |  |
| Serous borderline | 6(1.3%) | 1(0.3%) | 5(4.1%) |
| Mucinous borderline | 3(0.7%) | 2(0.6%) | 1(0.8%) |
| **Malignant tumors** |  |  |  |
| Serous cystadenocarcinoma | 28(6.1%) | 10(3.0%) | 18(14.9%) |
| Mucinous cystadenocarcinoma | 14(3.1%) | 6(1.8%) | 8(6.6%) |
| Secondary metastases to ovary | 6(1.3%) | 2(0.6%) | 4(3.3%) |
| Endometrioid carcinoma | 5(1.1%) | 4(1.2%) | 1(0.8%) |
| Clear cell carcinoma | 3(0.7%) | 0(0) | 3(2.5%) |
| Granulosa cell tumor | 3(0.7%) | 1(0.3%) | 2(1.7%) |
| Ovarian carcinosarcoma | 1(0.2%) | 1(0.3%) | 0(0) |
| Immature teratoma | 1(0.2%) | 1(0.3%) | 0(0) |
| Dysgerminoma | 1(0.2%) | 0(0) | 1(0.8%) |

^a^Other benign lesions included follicular cysts and luteinized follicle cysts, rete cystadenomas, inclusion cysts, stromal hyperplasia and hyperthecosis, Leydig cell hyperplasia, and lymphangioma.

| **eTable 5. Observed Frequencies of Different Tumor Types per Each Model’s Risk Categories^a^** | | | |
| --- | --- | --- | --- |
| **Risk model** | **Total No.** | **Histology confirmed benign lesion No. (%)** | **Histology confirmed malignant lesion No. (%)** |
| **Simple Rules** | | | |
| Simple Rules benign | 369 | 366(99.2%) | 3(0.8%) |
| Simple Rules malignant | 90 | 22(24.4%) | 68(75.6%) |
| **Assessment of Different Neoplasias in the ADNEX model^b^** | | | |
| < 1% | 4 | 4(100%) | 0(0) |
| < 3% | 159 | 158(99.4%) | 1(0.6%) |
| < 5% | 299 | 297(99.3%) | 2(0.7%) |
| < 10% | 347 | 341(98.3%) | 6(1.7%) |
| < 15% | 358 | 350(97.8%) | 8(2.2%) |
| **Ovarian-Adnexal Reporting and Data System (O-RADS) model^c^** | | | |
| O-RADS score 2 | 50 | 49(98.0%) | 1(2.0%) |
| O-RADS score 3 | 291 | 290(99.7%) | 1(0.3%) |
| O-RADS score 4 | 67 | 46(68.6%) | 21(31.3%) |
| O-RADS score 5 | 51 | 3(5.8%) | 48(94.1%) |

^a^Percentages reported in the table are row percentages.

^b^The median (interquartile range, IQR) risk of malignant tumor calculated by the ADNEX model was (3.3%; IQR, 2.4%-4.7%) for benign lesions and (53.6%; IQR, 25.1%-85.1%) for malignant lesions (P < 0.001, Mann-Whitney U test).

^c^The O-RADS model11 includes risk categories 0-5; scores 0-1 were not included in our study (score 0 for incomplete evaluation and score 1 for normal ovary including physiologic cyst).

| **eTable 6. Diagnostic Performance of the ADNEX Model at Different Thresholds for the Risk of Malignant Tumor Among 459 Adnexal Masses** | | | | |
| --- | --- | --- | --- | --- |
| **Threshold** | **Sensitivity,**  **% (95% CI)** | **Specificity,**  **% (95% CI)** | **PPV,**  **% (95% CI)** | **NPV,**  **% (95% CI)** |
| **Assessment of Different Neoplasias in the ADNEX model** | | | | |
| >=1% | 100(94.9-100) | 1.3(0.4-3.0) | 15.6(12.4-19.4) | 100(39.8-100) |
| >=3% | 98.6(92.4-100) | 43.6(38.6-48.7) | 24.2(19.4-29.6) | 99.4(96.8-100) |
| >=5% | 97.2(90.2-99.7) | 77.3(72.8-81.4) | 43.9(36.0-52.1) | 99.3(97.6-99.9) |
| >=10% | 91.6(82.5-96.8) | 88.1(84.5-91.2) | 58.6(48.8-67.8) | 98.3(96.3-99.4) |
| >=15% | 88.7(79.0-95.0) | 90.7(87.4-93.4) | 63.6(53.4-73.1) | 97.8(95.7-99.0) |

Abbreviations: ADNEX, Assessment of Different NEoplasias in the adneXa; NPV indicates Negative predictive value; PPV indicates Positive predictive value.

| **eTable 7. Malignant Frequencies per O-RADS Risk Scores When Stratified by the ADNEX and O-RADS Models^a^** | | |
| --- | --- | --- |
| **Risk model** | **Total No.** | **Histology confirmed malignant lesion No. (%)** |
| **Assessment of Different Neoplasias in the ADNEX model** | | |
| < 1% | 4 | 0(0) |
| 1-10% | 343 | 6(1.7%) |
| 10-50% | 68 | 27(39.7%) |
| >50% | 44 | 38(86.3%) |
| **Ovarian-Adnexal Reporting and Data System (O-RADS) model** | | |
| O-RADS score 2 | 50 | 1(2.0%) |
| O-RADS score 3 | 291 | 1(0.3%) |
| O-RADS score 4 | 67 | 21(31.3%) |
| O-RADS score 5 | 51 | 48(94.1%) |

^a^Secondary analysis, converting the continuous risk of the ADNEX model into discrete ordinal categories comparable to the O-RADS scores of 2-5. The table shows the observed malignant tumor prevalence per each risk score using the ADNEX and the O-RADS models to stratify patients into the risk groups.

Abbreviations: ADNEX, Assessment of Different NEoplasias in the adneXa; O-RADS, Ovarian-Adnexal Reporting and Data System.

| **eTable 8. Diagnostic performance of CA125, HE4, O-RADS, ADNEX and O-RADS combined with CA125 and HE4 in premenopausal and postmenopausal subgroups.** | | | | | | |
| --- | --- | --- | --- | --- | --- | --- |
| **Assessment methods** | **CA-125 (n=459)** | **HE4**  **(n=358)** | **ADNEX model**  **(n=459)** | **O-RADS model**  **(n=459)** | **O-RADS model + HE4**  **(n=358)** | **O-RADS model + CA-125 + HE4 (n=358)** |
| AUC (95% CI) | 0.58(0.53-0.63) | 0.75(0.70-0.79) | 0.95(0.93-0.97) | 0.96(0.94-0.97) | 0.96(0.94-0.97) | 0.96(0.94-0.97) |
| Sensitivity, % (95% CI) | 22.9(13.7-34.4) | 71.0(58.1-81.8) | 91.6(82.5-96.8) | 97.2(90.2-99.7) | 97.2(90.2-99.7) | 97.2(90.2-99.7) |
| Specificity, % (95% CI) | 94.3(91.5-96.4) | 76.4(71.1-81.1) | 88.1(84.5-91.2) | 87.4(83.6-90.5) | 87.4(83.6-90.5) | 87.4(83.6-90.5) |
| PPV, % (95% CI) | 42.1(26.1-59.4) | 38.6(29.6-48.2) | 58.6(48.8-67.8) | 58.5(49.0-67.5) | 58.5(49.0-67.5) | 58.5(49.0-67.5) |
| NPV, % (95% CI) | 87.1(83.5-90.2) | 92.6(88.6-95.6) | 98.3(96.3-99.4) | 99.4(97.9-99.9) | 99.4(97.9-99.9) | 99.4(97.9-99.9) |
| Positive LR (95% CI) | 4.0(2.6-6.2) | 2.7(2.2-3.3) | 7.7(7.1-8.4) | 7.7(7.3-8.1) | 7.7(7.3-8.1) | 7.7(7.3-8.1) |
| Negative LR (95% CI) | 0.8(0.5-1.3) | 0.5(0.3-0.7) | 0.1(0.04-0.2) | 0.03(0.008-0.1) | 0.03(0.008-0.1) | 0.03(0.008-0.1) |
| **Premenopausal** | **n=338** | **n=257** | **n=338** | **n=338** | **n=257** | **n=257** |
| AUC (95% CI) | 0.54(0.48-0.59) | 0.66(0.60-0.72) | 0.93(0.90-0.95) | 0.93(0.90-0.95) | 0.93(0.90-0.95) | 0.93(0.90-0.95) |
| Sensitivity, % (95% CI) | 25.0(10.7-44.9) | 54.2(32.8-74.4) | 85.7(67.3-96.0) | 92.9(76.5-99.1) | 92.9(76.5-99.1) | 92.9(76.5-99.1) |
| Specificity, % (95% CI) | 94.2(90.9-96.5) | 82.8(77.4-87.4) | 90.6(86.8-93.6) | 88.7(94.6-92.0) | 88.7(94.6-92.0) | 88.7(84.6-92.0) |
| PPV, % (95% CI) | 28.0(11.8-49.9) | 24.5(13.8-38.3) | 45.3(31.4-59.7) | 42.6(30.0-55.9) | 42.6(30.0-55.9) | 42.6(30.0-55.9) |
| NPV, % (95% CI) | 93.3(89.9-95.8) | 94.6(90.6-97.3) | 98.6(96.4-99.6) | 99.3(97.4-99.9) | 99.3(97.4-99.9) | 99.3(97.4-99.9) |
| Positive LR (95% CI) | 4.3(2.3-8.2) | 3.2(2.2-4.6) | 9.2(7.8-10.7) | 8.2(7.4-9.2) | 8.2(7.4-9.2) | 8.2(7.4-9.2) |
| Negative LR (95% CI) | 0.8(0.5-1.3) | 0.6(0.4-1.0) | 0.16(0.06-0.4) | 0.08(0.02-0.3) | 0.08(0.02-0.3) | 0.08(0.02-0.3) |
| **postmenopausal** | **n=121** | **n=101** | **n=121** | **n=121** | **n=101** | **n=101** |
| AUC (95% CI) | 0.76(0.67-0.83) | 0.73(0.63-0.81) | 0.95(0.89-0.98) | 0.96(0.91-0.99) | 0.96(0.91-0.99) | 0.96(0.91-0.99) |
| Sensitivity, % (95% CI) | 76.2(60.5-87.9) | 68.4(51.3-82.5) | 95.3(94.2-99.4) | 100(91.8-100) | 100(91.8-100) | 100(91.8-100) |
| Specificity, % (95% CI) | 70.5(59.1-80.3) | 74.6(62.1-84.7) | 79.5(68.8-87.8) | 82.1(71.7-89.8) | 82.1(71.7-89.8) | 82.1(71.7-89.8) |
| PPV, % (95% CI) | 58.2(44.0-71.5) | 61.9(45.6-76.4) | 71.9(58.5-83.0) | 75.4(62.2-85.9) | 75.4(62.2-85.9) | 75.4(62.2-85.9) |
| NPV, % (95% CI) | 84.6(73.5-92.4) | 79.7(67.2-89.0) | 96.9(89.1-99.6) | 100(94.4-100) | 100(94.4-100) | 100(94.4-100) |
| Positive LR (95% CI) | 2.6(2.1-3.2) | 2.7(2.1-3.5) | 4.7(4.1-5.3) | 5.6(5.0-6.2) | 5.6(5.0-6.2) | 5.6(5.0-6.2) |
| Negative LR (95% CI) | 0.3(0.2-0.6) | 0.4(0.2-0.8) | 0.06(0.01-0.2) | 0 | 0 | 0 |

Abbreviations: ADNEX, Assessment of Different Neoplasias in the Adnexa; LR, likelihood ratio; NPV, negative predictive value; O-RADS, Ovarian-Adnexal Reporting and Data System; PPV, positive predictive value. CA-125, Carbohydrate Antigen 125; HE4, Human Epididymis Protein 4
